# An Efficient Method for In Vitro Shoot-Tip Culture and Sporophyte Production Using *Selaginella martensii* Spring Sporophyte

**DOI:** 10.3390/plants9020235

**Published:** 2020-02-12

**Authors:** Kyungtae Park, Bo Kook Jang, Ha Min Lee, Ju Sung Cho, Cheol Hee Lee

**Affiliations:** 1 Division of Animal, Horticultural and Food Sciences, Chungbuk National University, Cheongju 28644, Korea; 2 Brain Korea 21 Center for Bio-Resource Development, Chungbuk National University, Cheongju 28644, Korea

**Keywords:** angle meristem, frosty fern, ornamental fern, rhizophore (leafless aerial root), Selaginellaceae

## Abstract

*Selaginella martensii*, an evergreen perennial fern that is native to South America and New Zealand, is named “frosty fern” because of its beautiful white-colored leaves and it is used as an ornamental plant. Efficient propagation methods for this species have not been developed. We aimed to develop an efficient propagation method for *S. martensii* through in vitro culture. We investigated culture conditions that are suitable for shoot-tip proliferation and growth. The optimum shoot-tip culture conditions were determined while using Murashige and Skoog (MS) medium (quarter, half, full, or double strength) and macronutrients (sucrose and two nitrogen sources) at various concentrations. In MS medium, the shoot tips formed a maximum of 6.77 nodes per explant, and each node formed two new shoot tips (i.e., 2^6^ or 64 shoot tips). When using branching segments containing an angle meristem, the shoot-to-rhizophore formation ratio could be controlled by medium supplementation with plant-growth regulators. Sporophytes that were grown from shoot tips in vitro were acclimated in *ex vitro* soil conditions and successfully survived in the greenhouse. Numerous shoot tips could be obtained from in vitro-grown sporophytes and be proliferated *ex vitro* to produce a large number of plants. This method provides a way of shortening the time that is required for producing a large stock of *S. martensii* planting material.

## 1. Introduction

*Selaginella martensii* Spring is an evergreen perennial fern that is native to South America and New Zealand that can form rhizophores (leafless aerial roots) at branching sites. It is named “frosty fern” because of its beautiful white leaves, which is also why it is used for ornamental purposes. Morphologically, two ventral and dorsal leaves are paired, and the stem is repeatedly branching and grown. In the angle meristem at the nodes, a rhizophore, a leafless cylindrical organ, can emerge and elongate, forming a root at the end of the rhizophore after growth [1]. In addition, a shoot can be formed when the rhizophore is separated from the mother plant or externally damaged [2,3].

*Selaginella* plants are heterosporous ferns that separately form megaspores and microspores, and gametophytes also exist in two types [4]. The germination timing of the two types of spores must match for heterosporous ferns to produce gametophytes. Yu [5] reported that the germination time of megaspores and microspores of *S. eremophila*, *S. rupincola,* and *S. arizonica* ranged from 10 to 20 and five to seven days, respectively. In addition, heterosporous ferns develop significantly slower than homosporous fern, even when gametophytes are developed after germination. The final fertilization rate was approximately 12% in *S. eremophila* and *S. rupincola*, and there was no fertilization in *S. arizonica* [5]. Therefore, the fertilization is rate is lower than that of homosporous ferns. For this reason, *S. martensii* is mainly propagated by the conventional cutting method while using leaves; however, propagation is time-consuming because of the low growth rate. This problem can be solved by producing sporophytes in vitro; however, studies on in vitro culture of *S. martensii* are scarce.

In vitro culture allows for producing uniform plants quickly and year-round in a controlled environment [6]. In addition, plants that are difficult to propagate in vivo can be propagated through various culture methods and be used for species conservation [7,8]. Homosporous ferns can be easily produced in vitro while using spores, gametophytes, and sporophytes, and various culture methods for these ferns have been successfully developed. Chopping and blending methods use mechanical fragmenting and the proliferation of prothalli from spores to obtain a large number of plants in a short time [9,10]. In vitro culture methods can induce regeneration and redifferentiation from prothalli to form somatic embryos, callus, and green globular bodies, or to obtain new plants from sporophyte tissues, including the leaf, rhizome, and stem [11,12,13,14,15,16,17]. In this study, we used *S. martensii* sporophytes to determine the explant types that are effective for in vitro propagation, and we optimized the culture medium composition for propagation and regeneration. In addition, we were able to produce a large number of plants in *ex vitro* soil conditions while, simultaneously, acclimating the plants by using sporophytes grown in vitro as a starting material. This new method increases the effectiveness and efficiency of *S. martensii* propagation as compared to existing protocols and it can be applied to other *Selaginella* species.

## 2. Results

### 2.1. Selection of Explant Type

**Figure 1:**
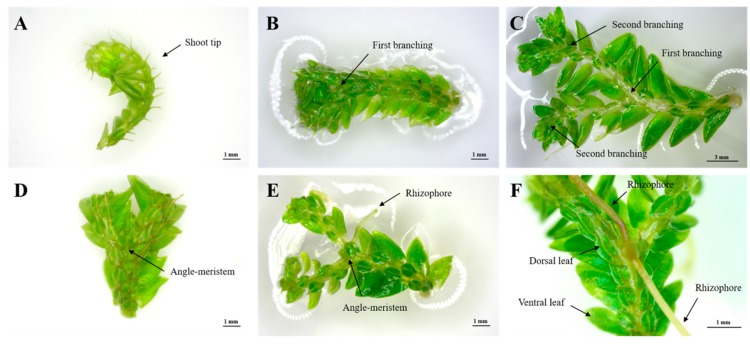
Explant types of *Selaginella martensii* Spring used in the experiments. (**A**) shoot tip, (**B**) first branching from shoot tip, (**C**) secondary branching, (**D**) branching segment, and (**E**,**F**) rhizophore developed in each of the two angle meristems.

Shoot tips (Figure 1A) and branching segments (Figure 1D) of *S. martensii* developed different organs in in vitro culture (Figure 1). The shoot-tip explant elongated, branched at the apex, and then formed two new shoot tips (Figure 1B). Thereafter, a number of new shoot tips were formed through repeated branching (Figure 1C). In contrast, branching segments only produced a rhizophore, without branching (Figure 1E). The angle meristem in the branching segment formed rhizophores in both the ventral and dorsal leaves, which developed into roots over time (Figure 1F). Based on these results, the shoot tip was selected as an optimal explant type, as it enabled branching and provides a continuous supply of explants.

### 2.2. Effect of Medium Components on Shoot-Tip Proliferation

**Figure 2:**
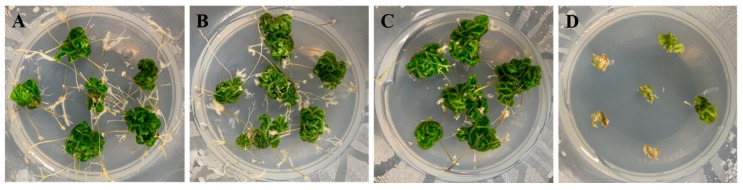
Growth response and organ formation of *Selaginella martensii* Spring explants cultured in different medium strength. (**A**–**D**), quarter-, half-, full-, and double-strength Murashige and Skoog (MS) media.

Shoot tips showed growth in Murashige and Skoog (MS) media of all strengths (Figure 2), but the explants showed browning in 2 MS medium. After 12 weeks of culture, in 1 MS medium, the fresh weight and the mean numbers of rhizophores were 0.68 g and 52.49, respectively, the mean maximum numbers of nodes were 6.77, and the mean total number of branches was 56.93 (Table 1). In 1/2 and 1/4 MS medium, the fresh weight was 0.40 and 0.22 g, respectively, and the number of rhizophores was 32.63 and 29.08, respectively. Therefore, further optimization of the medium was based on 1 MS medium.

**Figure 3:**
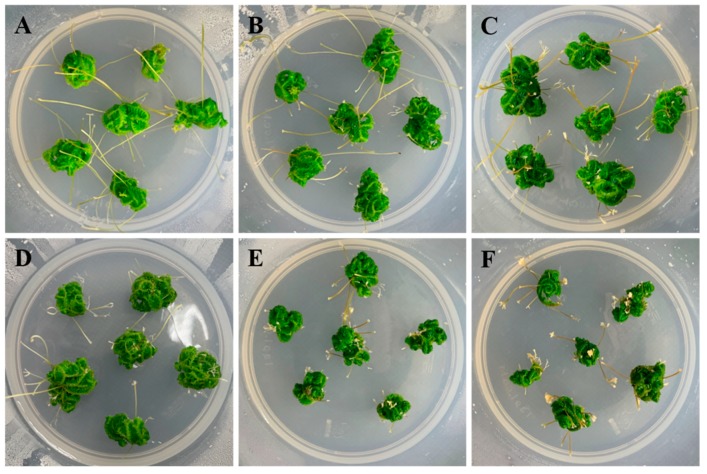
Growth response and organ formation of *Selaginella martensii* Spring explants cultured in 1 MS medium containing different concentration of sucrose. (**A**–**F**), 0, 1, 2, 3, 4, and 5% sucrose.

In 1 MS medium containing various concentrations of sucrose, explants developed normally, without browning or necrosis (Figure 3 and Table 2). Optimum growth was achieved in the presence of 2% sucrose. The mean fresh weight and rhizophore numbers were 0.54 g and 28.6, respectively, and the mean maximum numbers of nodes and total branches were 6.24 and 43.01, respectively. In medium containing 3–5% sucrose, the fresh weight (0.27–0.36 g) and rhizophore numbers (20.02–22.03) decreased. Growth was negatively affected. The explants could grow on medium without additional sucrose; however, all of the growth indicators had lower values in this condition.

**Figure 4:**
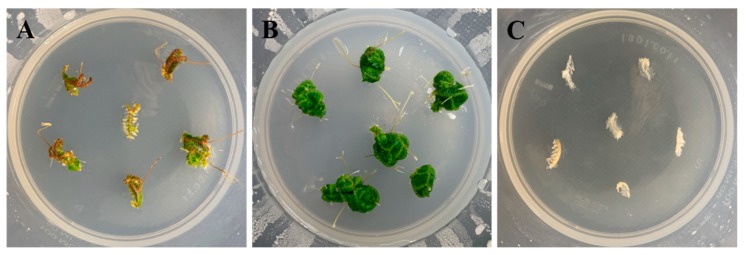
Growth response and organ formation of *Selaginella martensii* Spring explants cultured in 1 MS medium containing different total nitrogen concentration. (**A**–**C**), 30, 60, and 120 mM total nitrogen sources.

Table 3 shows the results for *S. martensii* shoot tips grown in 1 MS medium (3% sucrose) containing various concentrations of nitrogen (NH_4_Cl and KNO_3_). The shoot tips grew normally in medium containing 60 mM nitrogen. In medium that was supplemented with 120 mM nitrogen, the explants showed necrosis and, at 30 mM, they showed browning and poor growth (Figure 4). All of the shoot tips were necrotic in the medium that was supplemented with ammonium (NH_4_^+^) alone (Table 4). Significant differences in growth indicators were observed when the ratio of nitrate (NO_3_^–^) added to the medium was increased (NH_4_^+^:NO_3_^–^; 2:1, 1:1 and 1:2). In particular, there was an increase in the mean total number of branches (21.99–31.56) and the mean number of rhizophores (6.96–22.21). Supplementation of nitrate alone did not induce necrosis, but the shoot tips gradually browned and stopped growing.

### 2.3. Effects of Plant-Growth Regulators (PGRs) on the Induction of Branching Segments

**Figure 5:**
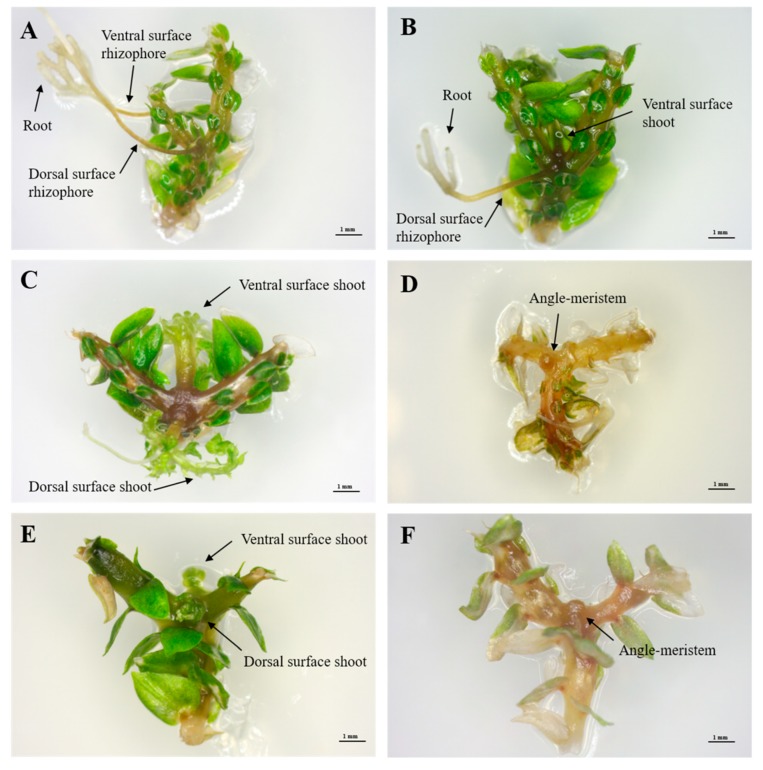
Organogenesis of branching segments of *Selaginella martensii* Spring cultured in medium supplemented with PGRs. (**A**) R/R (11.42 μM IAA), (**B**) S/R (2.46 μM IBA), (**C**) S/S (2.32 μM kinetin), (**D**) necrotic branching segment (9.30 μM kinetin), (**E**) S/S (2.22 μM BA), and (**F**) necrotic branching segment (4.44 μM BA). S/S, shoots; S/R, shoot and rhizophore; R/R, rhizophores formed from each angle meristem in the ventral and dorsal leaves.

Angle meristems in the ventral and dorsal leaves showed different responses, depending on the PGRs that were added to the MS medium (Table 5). In medium that was supplemented with 2.85 μM indole acetic acid (IAA), the angle meristems mainly formed shoot tips, and, as the concentration of IAA increased, rhizophore formation (and, thus, the rhizophore-to-shoot ratio) increased (Figure 5A). In medium that was supplemented with 2.46 μM indole butyric acid (IBA), 54.5% of the branching segments simultaneously formed shoot tips and rhizophores (Figure 5B). Rhizophore formation increased with increasing IBA concentration, as with IAA. In contrast, kinetin promoted shoot formation at all concentrations evaluated, except 9.30 μM (Figure 5C,D). 91.7% of explants formed shoots in medium that was supplemented with 2.22 μM of 6-benzylaminopurine (BA). As the BA concentration increased, the explants tended to necrotize (Figure 5E,F).

### 2.4. Ex Vitro Production of Sporophyte Seedlings

**Figure 6:**
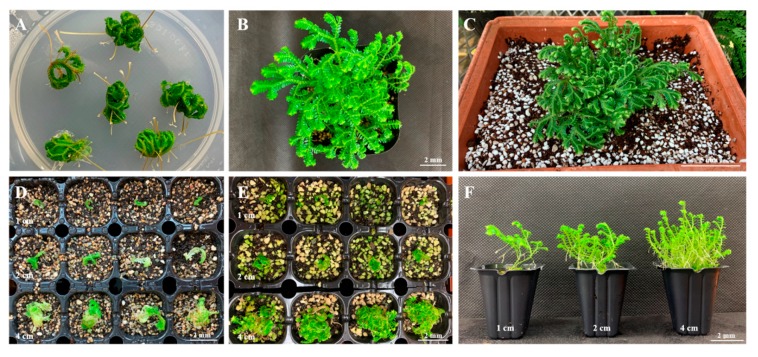
*Ex vitro* acclimation and propagation of whole plants and shoot cuttings from *S. martensii* Spring explants. (**A**) Whole plants obtained 12 weeks after culture (in MS medium). (**B**) Whole plants after eight weeks of acclimation under *ex vitro* conditions. (**C**) Plants grown in a glass greenhouse after acclimation. (**D**) Acclimation and propagation of shoots cut (1, 2, or 4 cm) from sporophytes in *ex vitro* conditions. (**E**,**F**) Plants that were obtained from shoots cut (1, 2, or 4 cm) from sporophytes after four and 12 weeks of acclimation and propagation.

Whole *S. martensii* plants (sporophytes) grown in vitro successfully survived in the glass greenhouse after *ex vitro* acclimation in soil for 12 weeks (Figure 6A–C). In addition, sporophytes that were cut at different lengths (1, 2, and 4 cm from the apex) were successfully acclimated and propagated in soil (Figure 6D–F). The survival rate of the cut sporophytes was 100% (data not shown). Sporophytes that were cut at 4 cm from the apex showed better overall growth than those cut at 1 or 2 cm from the apex as indicated by their higher fresh weight, dry weight, plant height, and root length (Table 6). We evaluated plant growth on horticultural substrate (Hs) alone or that mixed with decomposed granite (D) or perlite (Pr) at two different volume ratios. The plants grew normally on all five substrates. In particular, plants that were grown on the substrate mixtures showed significant differences in height (4 cm, Hs and D; 5.30–6.14 cm) and fresh weight (4 cm, Hs and D; 0.97–1.21 g), as the ratio of Hs increased in the order of Hs2 (2:1), Hs3 (3:1), and Hs1 (1:0). However, in the Hs and Pr mixtures, the growth parameters, including plant height (4.96–5.52 cm), root length (3.10–3.58 cm), and fresh (0.68–1.35 g) and dry weights (0.08–0.13 g), were lower than those in the other substrates. In particular, plant height (5.30–6.00 cm), root length (4.40–4.74 cm), and fresh (0.97–1.19 g) and dry weights (0.12–0.16 g) were higher in the Hs and D than in the other mixtures. *S. martensii* plants were successfully acclimated and they could survive in soil conditions, irrespective of the soil type and cutting length. However, after four weeks of ex vitro culture, significant differences in sporophyte growth were noted. In conclusion, it was most effective to cut and propagate *S. martensii* sporophyte to 4 cm in 3:1 mixture of Hs and D.

## 3. Discussion

Ferns can be cultured and propagated in vitro while using various explant types [17,18,19,20,21]. In this study, shoot tips of *S. martensii* successfully formed new branches, whereas branching segments only formed rhizophores. Angle meristems of cut branching segments mostly formed rhizophores, with a rhizophore-to-shoot ratio of 79%, according to Webster [22]. Intact-plant angle meristems mostly form rhizophores, but, when separated from the mother plant, they also formed shoots under the influence of endogenous hormones [2,22].

Shoot tips repeatedly branched during growth, followed by rhizophore formation. Next, roots were formed at the end of the rhizophore to absorb nutrients from the medium. As one shoot was transformed into a single plant, it was advantageous to propagate a large number of shoots from the new plant. The shoots were formed according to the number of nodes (Figure 1), and one shoot yielded approximately 16 shoots at the fourth branching frequency and 32 shoots at the fifth branching frequency, which was the maximum observed in this study. Thus, a large number of shoots were produced from shoot-tip explants. Therefore, and because branching segments only produced rhizophores, we selected shoot tips as an ideal explant.

MS medium is the most widely used medium for sporophyte culture of ferns [23]. Its nutritional composition can be adjusted to meet species-specific growth demands [11]. It is important to compose a species-specific medium suitable for plant growth, as salt and vitamin requirements for growth differ among species [21,22,23,24,25,26]. Jung and Lee [19] reported that sporophyte growth of *Dryopteris varia* was inhibited with decreasing MS medium strength. Low-strength MS is low in nutrients and, thus, nutrients are quickly depleted. High-strength MS is rich in nutrients, aiding plant growth [26], but plant growth might be inhibited due to osmotic stress that is caused by the high nutrient concentrations [27]. The total number of branches (56.93, 43.29, and 31.92) of *S. martensii* increased with increasing MS concentration (Table 1). Among them, 1 MS medium showed the most positive effect. In contrast, the high strength of 2 MS medium tended to strongly inhibit the formation of total number of branches (7.22) (Figure 2). *S. martensii* exhibited the effects similar to other ferns, and the media of various strengths were found to differentially affect growth.

In ferns, gametophyte and sporophyte production in vitro is affected by growth, which depends on the sucrose concentration [13]. Sucrose is used by the plants as an energy source for growth in vitro, and sucrose depletion leads to growth retardation and, in extreme cases, to growth inhibition and aging [28]. Rhizophores formed at angle meristems form a terminal root after elongation [1]. We found that *S. martensii* explants can survive and grow in medium without sucrose (Figure 3 and Table 2). However, growth was optimal in medium containing 2% sucrose, which indicated that sucrose is used as an energy source for growth by *S. martensii* plants. The amount and type of nitrogen that is required for growth in vitro is species dependent in ferns. In *Psilotum nudum* [29] and *Botrychium dissectum* [30], spore germination and plant growth are promoted in medium containing NH_4_^+^ nitrogen. In *Adiantum capillus-veneris*, sporophyte formation is promoted in medium containing NO_3_^–^ nitrogen [31]. Very low or high levels of nitrogen in the medium adversely affected the growth of plants (Figure 4 and Table 3). *Selaginella martensii* explants showed necrosis and growth inhibition in medium containing either NH_4_^+^ or NO_3_^–^ alone (Table 4). In addition, rhizophore growth tended to decrease when the NH_4_^+^:NO_3_^–^ ratio increased. Consistently, a high NH_4_^+^ concentration adversely affected gametophyte growth and morphogenesis in *Arachniodes aristata* and *Dryopteris nipponensis* [25,32]. In in vitro culture, NH_4_^+^ and NO_3_^–^ should both be added and, therefore, it is important to determine their optimum concentrations and ratio for different species [33].

In the current study, branching segments only formed rhizophores in the absence of exogenous PGRs. However, shoot formation from the angle meristem could be induced by the addition of exogenous PGRs to the medium (Figure 5). Cytokinins promoted cell division and play a decisive role in sporophyte formation in the propagation of ferns [34]. In particular, hormone BA affects bud formation and induced sporophyte production in this study [34]. In our study (Table 5), kinetin and BA induced new shoots from angle meristem, while inhibiting rhizophore formation. On the other hand, shoots and rhizophores were formed simultaneously in the presence of the auxin hormones IAA and IBA. The relation between sporophyte formation and auxin has been reported in several previous studies. IAA plays a role in inducing the regeneration of *Asplenium nidus* L. sporophytes [35]. Naphthalene acetic acid increases the number of leaves and sporophytes in *Asplenium septentrionale* and the number of fronds in *A. cuneifolium* [36]. Nevertheless, *S. martensii* only formed up to two shoots per branching-segment explant within the experimental period in this study, and growth and branching tended to be very slow when compared to those in shoot-tip explants. Therefore, branching segments were considered to be unsuitable for the formation of multiple shoots.

Acclimatization is required for plants grown in in vitro conditions to adapt to the external environment [37]. In vitro-produced sporophytes of *S. martensii* were successfully acclimated ex vitro and survived in a glass greenhouse (Figure 6). *S. martensii* plants that were exposed to the ex vitro environment rapidly formed multiple rhizophores at the angle meristems (Figure 6A–C), which seemed to be an adaptive response. In vitro-cultured *S. martensii* sporophytes were cut at different lengths during the acclimation process and then placed in the ex vitro soil condition. The cut explants showed strong rhizophore formation from the angle meristem, rooted well, and fully regenerated in the soil (Figure 6D–F). Thus, explants containing an angle meristem could be propagated ex vitro, eliminating the need for an intact sporophyte containing roots. The 4-cm-long explants contained more angle meristems than the 1- and 2-cm explants, resulting in better growth (Table 6). The ratio of Hs had no effect on the survival of cut sporophytes. In the subsequent growth stages, the growth parameters showed a significant difference as the ratio of Hs increased. This suggests that high nutrient levels are required after the formation of rhizophores. The multiple angle meristems rapidly formed rhizophores and they appeared to play a role in promoting plant growth. *Selaginella martensii* explants grew well ex vitro and successfully survived in the glass greenhouse. Therefore, it is more efficient to produce a large number of explants from a sporophyte and grow them ex vitro than to acclimate a sporophyte cultured in vitro. Thus, our study showed that this approach can increase *S. martensii* productivity. Previous studies have suggested regeneration methods for shoots while using PGRs [11,14,16], but our study revealed that the shoots were sufficiently regenerated without PGRs, allowing for direct *S. martensii* production.

## 4. Materials and Methods

### 4.1. Plant Material

The plant materials that were used in this study were fronds of *S. martensii* grown at the farm greenhouse of Chungbuk National University (Cheongju, Republic of Korea; 36°37′29.0″ N, 127°27′17.5″ E). Fronds were collected, washed with distilled water, and surface-sterilized in 70% ethanol for 1 min. Subsequently, the fronds were sterilized with 2% sodium hypochlorite for 15 min., washed with sterile water, and then incubated in MS medium (one 1-cm explant per 15-mL test tube) [38]. Primary-cultured plants were used as starting materials, and then subcultured at four-week intervals.

For in vitro culture, sporophyte shoot tips and branching segments were used (Figure 1). Shoot tips (approximately 12 mm in length) were cut from the top of the fronds and they did not include branching segments (including nodes). Branching segments containing the angle meristem, but without rhizophore formation were used to derive shoot tips from the angle meristem. The explants were incubated in Petri dishes containing MS medium (3% sucrose, 0.8% agar, pH 5.8) for eight weeks. All in vitro culture conditions were controlled at 25 ± 1 °C, a light intensity of 30 ± 1.0 PPFD (μmol m^–2^ s^–1^), and a 16-h light/8-h dark photoperiod.

### 4.2. Medium Optimization for Shoot-Tip Proliferation

MS medium was used at quarter, half, full, or double strength, and various nutrients were added at different concentrations, to select a medium suitable for shoot-tip propagation. Different levels of sucrose (0%, 1%, 2%, 3%, 4%, and 5%), total nitrogen sources (NH_4_Cl and KNO_3_, 1:2; 30, 60, or 120 mM), and NH_4_^+^:NO_3_^–^ ratios (3:0, 2:1, 1:1, 1:2, 0:3) were evaluated in full-strength MS. We used 120 × 80 mm culture vessels (Cat. No. 310120; SPL Life Sciences, Pocheon, Korea) containing 50 mL of medium each. After 12 weeks of culture, fresh weight per explant and the numbers of rhizophores, maximum nodes, and total branching points were determined.

### 4.3. Effects of PGRs on the Induction of Branching Segments

PGRs were added to MS medium (in Petri dishes) to induce shoot tips from the angle meristem. IAA (2.85, 5.71, 11.42 μM; Cas. No. 87-51-4), IBA (2.46, 4.92, 9.84 μM; Cat. No. 133-32-4), BA (2.22, 4.44, 8.88 μM; Cas. No. 1214-39-7), and kinetin (2.32, 4.65, 9.30 μM; Cas. No. 525-79-1) (all from Sigma–Aldrich) were added to MS medium (3% sucrose, NH_4_Cl and KNO_3_, 1:2; 60 mM, 0.8% agar, pH 5.8). Ten explants were used per treatment, and the organs that formed after eight weeks were examined under a stereoscopic microscope (SZ61; Olympus, Tokyo, Japan). Images of the organs were captured while using a CMOS camera (eXcope F630; Dixi Sci., Daejeon, Korea) and analyzed using eXcope 3.7.12277 software.

### 4.4. Evaluation of Sporophyte Seedling Growth in ex Vitro Conditions

Sporophytes grown in vitro were cut to various lengths (1, 2, and 4 cm from the apex) and grown *ex vitro* to produce a large number of sporophytes. In vitro plants were obtained from explants cultured for 12 weeks in MS medium (3% sucrose, NH_4_Cl and KNO_3_, 1:2; 60 mM, 0.8% agar, pH 5.8). Propagation and acclimation were carried out simultaneously. All of the sporophytes were exposed at 25 ± 1 °C by removing the lid of the culture vessel for 24 h prior to the transfer. Five soil mixtures were prepared while using horticultural substrate (Hs, Hanareum no. 2; Shinsung Mineral Co., Ltd., Goesan, Korea), decomposed granite (D, 2 mm; Samgye Masato, Gimhae, Korea), and perlite (Pr, Newpershine no. 2; GFC Co., Ltd., Hongseong, Korea). The following five soil mixtures were evaluated: Hs alone, 2:1 and 3:1 (*v/v*) mixtures of Hs and D, and 2:1 and 3:1 (*v/v*) mixtures of Hs and Pr. The sporophyte cuttings were sown in a plug tray (72 square cells; Bumnong, Co., Ltd., Jeongeup, Korea) containing each of the mixed soils, and the tray was placed in a plastic box (503 mm × 335 mm × 195 mm, SPC532; SH Plastic, Gyeongsan, Korea) and then covered with a glass plate for acclimation and growth while maintaining a relatively constant humidity (85% ± 5%). Plant height, root length, fresh weight, and dry weight were determined after 12 weeks of cultivation. The *ex vitro* condition was 25 ± 1 °C, a light intensity of 43 ± 2.0 PPFD (μmol·m^–2^·s^–1^), and a 16-h light/8-h dark photoperiod.

### 4.5. Data Analysis

All of the experiments were performed in a completely randomized design. All in vitro experiments included six explants per replicate, and four replicates were investigated. The ex vitro experiments included ten explants per treatment. SAS version 9.4 (SAS Institute Inc., Cary, NC, USA) was used to calculate the mean ± standard error for each treatment, and a factorial analysis was performed while using Duncan’s multiple range test, with a significance level of *p* < 0.05.

## 5. Conclusions

We suggested an optimized growth medium composition that can be used for in vitro culture of *S. martensii*. In MS medium, shoot tips formed numerous branches (nodes), with a maximum of 6.77 nodes per explant. When using branching segments containing an angle meristem, the shoot-to-rhizophore formation ratio could be controlled by medium supplementation with PGRs. Sporophytes that were grown in vitro successfully survived in soil conditions, cuttings from in vitro -grown sporophytes could be directly propagated in soil conditions. This approach provides a way of shortening the time that is required for producing a large stock of *S. martensii* planting material.

## Figures and Tables

**Table 1:** Effect of culture medium strength on growth of *Selaginella martensii* Spring after 12 weeks of culture.

Medium Strength	Fresh Weight/Explant (g)	Maximum No. of Nodes/Explants	Total No. of Branches/Explant	No. of Rhizophores/Explant
1/4 MS	0.22 ± 0.02 c	5.63 ± 0.08 b	31.92 ± 1.70 c	29.08 ± 1.06 b
1/2 MS	0.40 ± 0.02 b	5.92 ± 0.21 b	43.29 ± 5.26 b	32.63 ± 2.70 b
1 MS	0.68 ± 0.06 a	6.77 ± 0.35 a	56.93 ± 3.00 a	52.49 ± 4.77 a
2 MS	0.15 ± 0.04 c	2.78 ± 0.22 c	7.22 ± 1.13 d	2.22 ± 0.84 c

Different letters within columns indicate a significant difference by Duncan’s multiple range test at the 5% level.

**Table 2:** Effect of sucrose concentration on growth of *Selaginella martensii* Spring after 12 weeks of culture.

Sucrose (%)	Fresh Weight/Explant (g)	Maximum No. of Nodes/Explant	Total No. of Branches/Explant	No. of Rhizophores/Explants
0	0.21 ± 0.01 c	4.96 ± 0.14 b	13.79 ± 0.92 c	11.17 ± 0.91 c
1	0.35 ± 0.06 b	5.31 ± 0.32 b	26.24 ± 3.39 b	22.60 ± 1.91 ab
2	0.54 ± 0.05 a	6.24 ± 0.39 a	43.01 ± 7.17 a	28.60 ± 4.01 a
3	0.36 ± 0.05 b	5.52 ± 0.29 ab	31.29 ± 3.23 b	22.03 ± 1.67 ab
4	0.29 ± 0.01 bc	5.15 ± 0.15 b	24.13 ± 1.57 bc	20.02 ± 1.45 b
5	0.27 ± 0.01 bc	5.14 ± 0.21 b	24.16 ± 1.69 bc	21.12 ± 1.21 b

Different letters within columns indicate a significant difference by Duncan’s multiple range test at the 5% level.

**Table 3:** Effect of total nitrogen concentration on growth of *Selaginella martensii* Spring after 12 weeks of culture.

Total Nitrogen Conc. (mM)	Fresh Weight/Explant (g)	Maximum No. of Nodes/Explant	Total no. of Branches/Explant	No. of Rhizophores/Explants
30	0.04 ± 0.01 b ^z^	1.72 ± 0.24 b	1.83 ± 0.29 b	2.00 ± 0.17 b
60	0.17 ± 0.05 a	5.00 ± 0.20 a	20.63 ± 0.98 a	16.13 ± 1.09 a
120	0.01 ± 0.00 c	0.00 c	0.00 c	0.00 c

Different letters within columns indicate a significant difference by Duncan’s multiple range test at the 5% level.

**Table 4:** Effect of ammonium-to-nitrate nitrogen ratio on growth of *Selaginella martensii* Spring after 12 weeks of culture.

NH_4_^+^:NO_3_^–^	Fresh Weight/Explant (g)	Maximum No. of Nodes/Explant	Total No. of Branches/Explant	No. of Rhizophores/Explants
3:0	0.01 ± 0.00 c	0.00 c	0.00 c	0.00 d
2:1	0.33 ± 0.02 a	5.39 ± 0.37 a	21.99 ± 1.65 b	6.96 ± 0.70 c
1:1	0.36 ± 0.02 a	5.85 ± 0.32 a	28.81 ± 1.10 a	13.95 ± 2.85 b
1:2	0.37 ± 0.04 a	5.51 ± 0.34 a	31.56 ± 3.67 a	22.21 ± 3.28 a
0:3	0.14 ± 0.05 b	2.58 ± 0.25 b	5.25 ± 0.08 c	5.57 ± 0.23 cd

Different letters within columns indicate a significant difference by Duncan’s multiple range test at the 5% level.

**Table 5:** Effects of PGRs and concentrations on angle-meristem development and organogenesis of branching segments in *Selaginella martensii* Spring.

PGRs	Concentration (μM)	Survival Rate (%)	Organs Formed in Angle Meristem (%)		
			S/S	S/R	R/R
	0.00	100.0	0.0	0.0	100.0
IAA	2.85	91.7	72.7	18.2	9.1
	5.71	100.0	0.0	16.7	83.3
	11.42	100.0	8.3	8.3	83.3
IBA	2.46	91.7	18.2	54.5	27.3
	4.92	100.0	16.7	8.3	75.0
	9.84	100.0	0.0	0.0	100.0
Kinetin	2.32	100.0	100.0	0.0	0.0
	4.65	100.0	100.0	0.0	0.0
	9.30	0.0	0.0	0.0	0.0
BA	2.22	100.0	91.7	8.3	0.0
	4.44	0.0	0.0	0.0	0.0
	8.88	0.0	0.0	0.0	0.0

S/S, shoots; S/R, shoot and rhizophore; R/R, rhizophores formed from each angle meristem in the ventral and dorsal leaves.

**Table 6:** Effect of soil substrate and cutting length on growth of *Selaginella martensii* Spring after 12 weeks of acclimation and propagation in *ex vitro* conditions.

Substrate	Length (cm)	Plant Height (cm)	Root Length (cm)	Fresh Weight (g)	Dry Weight (g)
Hs1	1	5.50 ± 0.38 a–d	2.78 ± 0.22 c–e	0.28 ± 0.10 d	0.03 ± 0.01 ef
	2	5.82 ± 0.38 a-c	4.30 ± 0.32 ab	0.40 ± 0.06 d	0.06 ± 0.01 dc
	4	6.14 ± 0.56 a	4.54 ± 0.29 a	1.21 ± 0.18 ab	0.13 ± 0.02 ab
Hs2-D1	1	4.54 ± 0.26 de	3.64 ± 0.25 bc	0.29 ± 0.06 d	0.04 ± 0.01 d-f
	2	4.80 ± 0.31 c-e	3.50 ± 0.33 bc	0.31 ± 0.04 d	0.04 ± 0.01 d-f
	4	5.30 ± 0.31 a-d	4.74 ± 0.50 a	0.97 ± 0.13 b	0.12 ± 0.01 b
Hs3-D1	1	5.66 ± 0.45 a-d	3.60 ± 0.22 bc	0.19 ± 0.06 d	0.05 ± 0.01 de
	2	4.94 ± 0.18 b-e	4.32 ± 0.18 ab	0.26 ± 0.03 d	0.04 ± 0.00 d-f
	4	6.00 ± 0.37 ab	4.40 ± 0.34 ab	1.19 ± 0.08 ab	0.16 ± 0.00 a
Hs2-Pr1	1	5.20 ± 0.19 a-d	2.14 ± 0.19 e	0.21 ± 0.02 d	0.02 ± 0.00 ef
	2	4.54 ± 0.24 de	2.50 ± 0.09 de	0.23 ± 0.04 d	0.03 ± 0.00 ef
	4	4.96 ± 0.27 b-e	3.10 ± 0.28 cd	0.68 ± 0.07 c	0.08 ± 0.01 c
Hs3-Pr1	1	5.08 ± 0.29 a-e	2.12 ± 0.26 e	0.18 ± 0.05 d	0.02 ± 0.00 f
	2	4.06 ± 0.36 e	2.32 ± 0.36 de	0.25 ± 0.05 d	0.03 ± 0.01 ef
	4	5.52 ± 0.42 a-e	3.58 ± 0.07 bc	1.35 ± 0.10 a	0.13 ± 0.01 b
Significance					
A (substrate)		**	***	**	***
B (length)		**	***	***	***
A × B		NS	NS	*	*

Different letters within columns indicate a significant difference by Duncan’s multiple range test at the 5% level. Hs, horticultural substrate; D, decomposed granite; Pr, perlite; substrate numbers 1, 2, or 3 represents volume ratio of each soil. NS, * *p* < 0.05, ** *p* < 0.01, and *** *p* < 0.001.
